# Outbreak of hantavirus disease caused by Puumala virus, Croatia, 2021

**DOI:** 10.2807/1560-7917.ES.2025.30.3.2400127

**Published:** 2025-01-23

**Authors:** Mari Rončević Filipović, Zlatko Trobonjača, Đurđica Cekinović Grbeša, Marinko Filipović, Melita Kukuljan, Ena Mršić, Vanja Tešić, Stela Živčić-Ćosić

**Affiliations:** 1 Clinical Hospital Center Rijeka, Rijeka, Croatia; 2 Faculty of Medicine, University of Rijeka, Rijeka, Croatia; 3 Faculty of Health Studies, University of Rijeka, Rijeka, Croatia; 4 Teaching Institute of Public Health Dr. Andrija Štampar, Zagreb, Croatia

**Keywords:** COVID-19, epidemic nephropathy, hantavirus infection, haemorrhagic fever with renal syndrome, Puumala virus

## Abstract

In 2021, a large outbreak of hantavirus disease (HAVID) in Croatia with 334 notified cases coincided with a COVID-19 wave and included patients from areas previously not considered endemic, challenging HAVID recognition and patient management. We analysed clinical and epidemiological data on all 254 patients with HAVID treated in the Clinical Hospital Center Rijeka (CHC Rijeka) between February and November 2021. Most patients (n = 246; 96.9%) had antibodies against Puumala virus, 212 (83.5%) were residents of endemic areas for HAVID, 93 (36.6%) reported occupational exposure and 86 (33.9%) had observed rodents or rodent excreta. Thirty-seven (14.6%) patients were not notified to the public health authorities. Most patients (n = 177; 69.7%) were male. The median age of the patients was 43 years (range: 17–79 years) in males and 54 years (range: 14–77 years) in females. More severe courses of disease were observed in males aged < 45 years than in older males and females of any age (OR = 2.27; 95% CI: 1.21–4.24; p < 0.005). Measures to prevent exposure, early detection and notification of cases and close collaboration between primary and secondary healthcare teams with public health personnel are essential to improve surveillance and prevent hantavirus outbreaks.

**Key public health message**

**What did you want to address in this study and why?**

Hantaviruses, carried by rodents, can cause haemorrhagic fever with kidney failure in humans. In 2021, Croatia experienced one of the largest hantavirus disease outbreaks in the country, during a COVID-19 pandemic wave. We analysed clinical and epidemiological data of patients with hantavirus disease treated in a hospital in Croatia.

**What have we learnt from this study?**

Of the 254 patients with hantavirus disease, more than 80% were residents of endemic regions and more than a third were exposed to the virus at work. More severe courses of disease were observed in younger males. Not all patients with suspected hantavirus disease were notified to the authorities.

**What are the implications of your findings for public health?**

Healthcare professionals need to be aware of the importance of timely diagnosis and notification of hantavirus infections. More information is needed on how to protect persons living, working or visiting areas where hantaviruses are present, from transmission of the virus. Collaboration across disciplines and sectors is necessary to improve surveillance and prevent hantavirus outbreaks.

## Background

Viruses of the Orthohantavirus genus, family *Hantaviridae*, order *Elliovirales*, are the causative agents of three main clinical syndromes: epidemic nephropathy, haemorrhagic fever with renal syndrome (HFRS) and hantavirus cardiopulmonary syndrome (HCPS) [1,2]. Puumala virus, which is widespread in Europe, Dobrava virus occurring in south-eastern Europe and Seoul virus, present globally, cause HFRS and epidemic nephropathy. Andes virus, Sin Nombre virus and several other hantaviruses prevalent in the Americas cause HCPS [1,3].

In Croatia and the neighbouring countries of Slovenia and Bosnia and Herzegovina, hantavirus outbreaks are known to occur [1,4-7]. The main causative agents are Puumala virus and to a much lesser extent Dobrava virus. The bank vole (*Clethrionomys glareolus*) is the reservoir host of Puumala virus, and the yellow-necked mouse (*Apodemus flavicollis*) the reservoir host of Dobrava virus [8]. Increases in the incidence of hantavirus disease (HAVID) cases in humans are related to higher rodent densities. The mechanisms that drive hantavirus outbreaks are complex, multi-factorial and still largely unknown [9]. Environmental factors, such as warm and rainy weather with lush vegetation growth (mast phenomenon), and increased food availability lead to a higher reproduction of the rodent population [2,4,5,9]. Humans become infected through inhalation of aerosolised virus-containing particles or via direct or indirect contact with infected rodent excreta.

Between 2016 and 2020, according to data reported to European Centre for Disease Prevention and Control (ECDC), the overall notification rate of hantavirus infection fluctuated between 0.4 and 1.0 cases per 100,000 population in Europe and between 0.4 and 9.4 cases per 100,000 population in Croatia [1]. In Croatia, the first HAVID cases were diagnosed in the early 1950s [10]. Several enzootic areas have been described, primarily in the continental parts of the country. However, the complete geographical distribution of this zoonosis is unknown [11-13]. Hantaviruses have a high epidemic potential and represent a considerable public health threat [1,3].

## Outbreak detection

On 6 February 2021, a patient with fever, headache, lumbar pain, blurred vision, thrombocytopaenia, acute kidney injury and a mild elevation in transaminases, was admitted to the Clinical Hospital Center Rijeka (CHC Rijeka), Croatia, and on 12 February, another patient with similar presentation sought healthcare. A suspicion of HAVID was raised. Both patients reported having symptoms for 1 week, noticing rodent infestations and considered the endemic regions of the north-western inland of Croatia, the Lika-Senj County and the Primorje-Gorski Kotar County, as the likely places of exposure. Antibodies (IgM and IgG) to Puumala virus were detected in blood samples of these two patients at the laboratory of the Croatian Institute of Public Health (CIPH). We received the test results of the index case on 25 February and of the other case on 15 March 2021.

Subsequently, in March 2021, 21 HAVID patients were treated in the CHC Rijeka, with 14 of them working for a forest company or in forests of the endemic regions of the Primorje-Gorski Kotar County and the Lika-Senj County, where HAVID cases have been notified almost every year. Blood samples from these patients were serologically tested at the CIPH and the University Hospital for Infectious Diseases in Zagreb. By grouping of these cases with clinical presentations and laboratory findings characteristic for HAVID and considering the epidemiological link, we suspected an outbreak. After receiving the serological testing results, we notified the Institute of Public Health (IPH).

Herein, we describe HAVID cases treated in the CHC Rijeka in 2021, with focus on clinical and epidemiological characteristics of the patients.

## Methods

### Surveillance of hantavirus disease in Croatia

In Croatia, the Division for Communicable Disease Epidemiology of the CIPH, in close cooperation with regional IPHs, is responsible for surveillance of infectious diseases and regional and national registries on infectious diseases. According to the Croatian legislation, HAVID is a mandatorily notifiable disease in humans and the treating physician or other healthcare worker must notify, as a rule, based on the clinical diagnosis within 24 h, while laboratory confirmation is subsequently notified upon receipt of the testing results [14]. Patients with suspected HAVID are usually referred to an infectious disease specialist and/or nephrologist, who are working at the hospitals and diagnosing the cases. The laboratories are not responsible for reporting the results. Sporadic cases are notified every year, and large outbreaks occur, the largest in 2017 with 389 notified cases, which prompted the introduction of a standardised questionnaire used by the IPHs [1,4]. Testing for antibodies against Puumala and Dobrava virus is the primary diagnostic method. Following the observation of a higher incidence of hantavirus infections, i.e., not only sporadic cases, the IPHs inform the general practitioner (GP) practices, hospitals, and if needed, pest control services. There is no defined threshold on the increased incidence of HAVID cases nor an automatic warning system.

### Definitions of cases and disease severity

The three categories of HAVID cases are described in Box [15].

Box **Criteria for categorisation of hantavirus cases, hantavirus outbreak, Croatia, 2021**

**Possible case:**

• *Clinical criteria:*

o Influenza-like illness, fever, headache, lumbar and/or abdominal pain, gastrointestinal symptoms, visual disturbances, conjunctival hyperaemia.

o Five phases in typical presentations: Febrile, hypotensive, oliguric, polyuric and recovery phases.

• *Laboratory findings:*

o Thrombocytopaenia^a^, proteinuria, microhaematuria, elevation of serum creatinine, C-reactive protein and/or transaminases.

**Probable case^b^:**

• *Possible case*

AND

• *Epidemiological criteria:*

o Environmental exposure, including inhalation, contact with or ingestion of contaminated materials during the incubation period.

**Confirmed case:**

• *Probable case*

AND

• *Laboratory criteria:*

o Detection of IgM and IgG antibodies to hantaviruses.

AND/OR

o Fourfold increase in hantavirus antibody titres in paired serum samples.

^a^ Thrombocytopaenia: thrombocyte count < 158 × 10^9^/L for patients aged > 19 years and < 178 × 10^9^/L for patients aged < 19 years, according to the reference values of Flegar-Meštrić et al. [17].^b^ Case definitions for notifiable diseases, Croatian Institute of Public Health [15].

We categorised HAVID as a mild to moderate or a severe disease, based on the clinical presentation. In mild to moderate disease, body temperature was < 40°C, and besides the common clinical criteria, this category also included patients with pleural effusion, bronchopneumonia without the need of oxygen supplementation and/or renal insufficiency without the need of acute dialysis. In severe disease, patients had at least one of the following: fever ≥ 40°C, bronchopneumonia with the need of oxygen supplementation, respiratory failure, heart failure, pulmonary oedema, acute dialysis treatment, haemodynamic instability, mental confusion, seizures, thrombotic or thromboembolic complications.

### Microbiological analyses

Blood samples from suspected cases were tested by ReaScan + PUUMALA IgM and ReaScan DOBRAVA-HANTAAN IgM immunochromatographic assays (Reagena, Toivola, Finland) at the laboratory of the University Hospital for Infectious Diseases in Zagreb or at the laboratory of the CIPH by an indirect immunofluorescence assay, Hantavirus Mosaic 1 (Euroimmun Medizinische Labordiagnostika AG, Lübeck, Germany), to detect IgM and IgG antibodies against Puumala, Dobrava, Hantaan, Saaremaa and Seoul viruses. A result of the immunofluorescence test was considered positive at a dilution of ≥ 1:100.

### Clinical and epidemiological data

We retrieved epidemiological data on HAVID from the notification system of communicable diseases of the CIPH, the regional IPHs and ECDC [1,4,16]. Routine data on all patients treated for probable and confirmed HAVID in the CHC Rijeka were collected from health records, extracted from the hospital digital database and pseudonymised.

We used the databases to analyse epidemiological data and patient characteristics, based on routine medical history obtained by the treating physicians, as well as laboratory findings, treatment data, disease complications and outcomes during the hospital treatment. No standardised questionnaire was used.

### Statistical analysis

We described participants with absolute numbers and proportions by sex, age, reported mode of exposure, the presence of comorbidities, laboratory findings, disease complications and hospital treatment. Additionally, we compared disease severity in patients by sex, age, comorbidity, reported mode of exposure, symptoms or signs of HAVID and laboratory parameters.

Participants with missing values in relevant variables were excluded from the calculations of proportions but we reported their absolute numbers. Age differences were expressed as the median ± range of the median because the normality assumption was violated (Kolmogorov-Smirnov test). We used Mann–Whitney U test for group comparisons by median age.

Fisher’s exact test was used for comparing the proportions of patients by disease severity according to abnormal laboratory findings. Logistic regression was applied to obtain odds ratios (OR) with 95% confidence intervals (CI). All statistical calculations were performed using TIBCO Data Science - Workbench (Statistica) version 14.1.0.8 (https://www.tibco.com/). Results with a p value < 0.05 were considered statistically significant.

## Results

### Descriptive epidemiology

**Figure 1:**
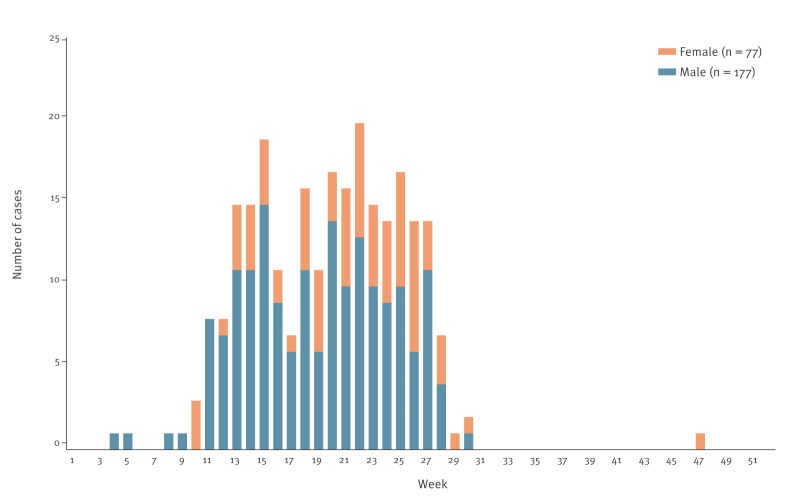
Timeline of cases treated for hantavirus disease, by week of disease onset and sex, Clinical Hospital Center Rijeka, Croatia, 2021 (n = 254)

Between February and July 2021, 255 patients, and in November 2021, one patient with suspected HAVID were treated in the CHC Rijeka. Of these, 246 were confirmed and eight were probable cases (Figure 1). Two cases with negative hantavirus antibody testing results were excluded from the analyses. Of the 254 cases included, 229 (90.2%) were from the Primorje-Gorski Kotar County and 25 (9.8%) from the Lika-Senj County. Thirty-seven (16.2%) of the patients from the Primorje-Gorski Kotar County were not notified to the IPH.

**Figure 2:**
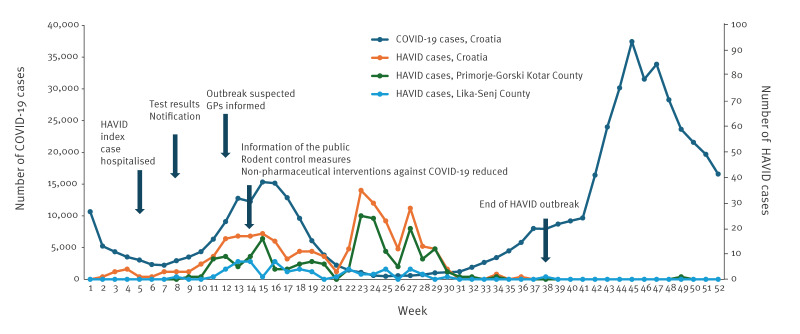
Number of cases of hantavirus disease (n = 334) and COVID-19 (n = 501,232) and response measures to hantavirus disease outbreak, by week, Croatia, 2021^a^

GPs: general practitioner practices; HAVID: hantavirus disease.

^a^ Data retrieved from the notification system of communicable diseases of the Division for Communicable Disease Epidemiology, Croatian Institute of Public Health.

The outbreak occurred during the third wave of the COVID-19 pandemic in our country (Figure 2) [4,16]. The Primorje-Gorski Kotar County was affected by both diseases, for instance, on 30–31 March 2021, 886 (21%) of all newly confirmed cases of COVID-19 reported in Croatia were from the Primorje-Gorski Kotar County which constitutes < 7% of the Croatian population [16].

Epidemiological data and baseline characteristics of the patients with HAVID, treated in the CHC Rijeka, are presented in Figure 1 and Table 1. Most patients were male (n = 177; 69.7%). Males aged < 45 years were more likely to be a HAVID patient than males aged ≥ 45 years (OR = 3.82; 95% CI: 2.07–7.06; p < 0.001). The median age of the patients with HAVID was 48 years (range: 14–79 years), lower in males (43 years; range: 17–79 years) than in females (54 years; range: 14–77 years) (p < 0.001). Of the 111 patients aged 45–64 years, 68 were males and 43 females, whereas of the 34 patients aged ≥ 65 years, 17 were males and 17 females (Table 1).

**Table 1:** Description of patients treated for hantavirus disease in the Clinical Hospital Center Rijeka, by age and mode of exposure, Croatia, 2021 (n = 254)^a^

Characteristics	Males n = 177		Females n = 77		All		OR		95% CI	p value
	n	%	n	%	n	%				
Age (years)										
< 45	92	52.0	17	22.1	109	42.9	3.82	2.07–7.06		< 0.001
45–64	68	38.4	43	55.8	111	43.7	0.49	0.29–0.85		0.005
≥ 65	17	9.6	17	22.1	34	13.4	0.38	0.18–0.78		0.004
Age (years) of patients with comorbidities (n = 93)										
< 45	18	31.6	6	16.7	24	25.8	2.31	0.82–6.53		0.057
45–64	27	47.4	16	44.4	43	46.2	1.13	0.49–2.60		0.391
≥ 65	12	21.1	14	38.9	26	28.0	0.42	0.17–1.06		0.033
Reported mode of exposure										
Workplace exposure only	18	10.2	1	1.3	19	7.5	8.60		1.13–65.65	0.019
Workplace exposure and residence	66	37.3	8	10.4	74	29.1	5.13		2.32–11.33	< 0.001
Residence only	79	44.6	59	76.6	138	54.3	0.25		0.13–0.45	< 0.001
Leisure activity	10	5.6	5	6.5	15	5.9	0.86		0.29–2.61	0.397
Unknown	4	2.3	4	5.2	8	3.2	0.42		0.10–1.73	0.116

CI: confidence interval; OR: odds ratio (for males in relation to females).

^a^ 246 confirmed and eight probable cases.

Major comorbidities included arterial hypertension (n = 47), cardiac disease (n = 19), diabetes mellitus (n = 13), bronchial asthma (n = 9), psychiatric disorder (n = 9), autoimmune disease (n = 7), cerebrovascular disease (n = 7), malignancy (n = 6), hypothyroidism (n = 6), obesity (n = 5), inflammatory bowel disease (n = 2), post-surgical abdominal adhesions (n = 2), prostatic hyperplasia (n = 2) and chronic gastritis, chronic hepatitis B, post-traumatic asplenia, multiple sclerosis, atopic dermatitis, osteoporosis in one patient each. Of the 26 patients aged ≥ 65 years with comorbidities, 14 were females (Table 1).

Of the 254 patients, 212 (83.5%) were residents of the endemic regions of the Primorje-Gorski Kotar County and the Lika-Senj County. Among the 93 (36.6%) patients reporting occupational exposure, 84 (90.3%) were male (e.g. foresters, workers in the wood industry, sawmills, construction, warehouses, farmers, cattle breeders, gamekeepers and hunters), while females were, more often, residents of endemic areas without an occupational exposure (Table 1). Among the 138 patients without a workplace exposure, 30 were from the coastal region and had no history of visits or other possible exposure in known endemic areas for HAVID during the incubation period. According to the medical records, 86 (33.9%) of the 254 patients had observed rodents or rodent excreta in the endemic region, mainly in the houses and the surroundings. However, we do not know how many patients were not asked about exposure to rodents because no standardised questionnaire was used. We did not find any data in the patient medical histories on prior hantavirus infections.

### Microbiological investigations

Antibodies against Puumala virus were detected in 246 (96.9%) of the 254 patients: the immunochromatographic assay was used for testing sera of 241 (98.0%) patients and the immunofluorescent test for sera of five (2.0%) patients. Samples from 40 patients were co-tested for IgM antibodies against Puumala and Dobrava virus. All 40 had IgM antibodies against Puumala virus, six had antibodies against Dobrava and eight had borderline levels against Dobrava virus. The antibody titres against Puumala virus were higher than those against Dobrava virus. No IgM antibodies against the other viruses were detected.

### Disease severity

Demographic data and reported mode of exposure are shown in Table 2. We compared disease severity in patients aged < 45, 45–64 and ≥ 65 years. The median age of male patients with a severe disease was 34 years (range: 17–79 years) and 47 years (range: 18–78 years) of males with mild to moderate disease. More severe cases of HAVID were observed in males aged < 45 years than in older males and females of any age (OR = 2.27; 95% CI: 1.21–4.24; p = 0.005). Comorbidities or mode of exposure did not have a significant effect on disease severity (Table 2).

**Table 2:** Disease severity of patients treated in the Clinical Hospital Center Rijeka for hantavirus disease, by age, sex and mode of exposure, Croatia, 2021 (n = 254)^a^

Characteristics	Severe disease n = 50		Mild to moderate disease n = 204		Total		p value^b^		
	Median age	Range	Median age	Range	Median age	Range			
Age (years)									
Males	34	17–79	47	18–78	44	17–79	0.041		
Females	59	14–77	55	19–74	56	14–77	0.490		
Males with comorbidity	45	17–79	57	23–78	56	17–79	0.535		
Females with comorbidity	65	14–77	60	34–74	60	28–77	0.704		
Age group (years)	n	%	N	%	n	%	OR	95% CI	p value
Males (n = 177)									
< 45	26	52.0	66	32.4	92	36.2	2.27	1.21–4.24	0.005
45–64	6	12.0	62	30.4	68	26.8	0.31	0.13–0.77	0.006
≥ 65	6	12.0	11	5.4	17	6.7	2.39	0.84–6.82	0.051
Females (n = 77)									
< 45	3	6.0	14	6.9	17	6.7	0.87	0.24–3.14	0.413
45–64	6	12.0	37	18.1	43	16.9	0.62	0.24–1.55	0.152
≥ 65	3	6.0	14	6.9	17	6.7	0.87	0.24–3.14	0.413
Comorbidity (n = 82)									
Males with comorbidity	12	24.0	36	17.6	48	18.9	1.47	0.70–3.10	0.153
Females with comorbidity	7	14.0	27	13.2	34	13.4	1.07	0.44–2.61	0.443
Reported mode of exposure									
Workplace exposure only	3	6.0	16	7.8	19	7.5	0.75	0.21–2.68	0.329
Workplace exposure and residence	16	32.0	58	28.4	74	29.1	1.18	0.61–2.31	0.309
Residence only	28	56.0	110	53.9	138	54.3	1.09	0.58–2.03	0.396
Leisure activity	2	4.0	13	6.5	15	5.9	0.61	0.13–2.81	0.264
Unknown	1	2.0	7	3.4	8	3.2	0.57	0.07–4.78	0.304

CI: confidence interval; OR: odds ratio (for severe disease in relation to mild to moderate disease).

^a^ 246 confirmed and eight probable cases.

^b^ Mann–Whitney U test.

**Figure 3:**
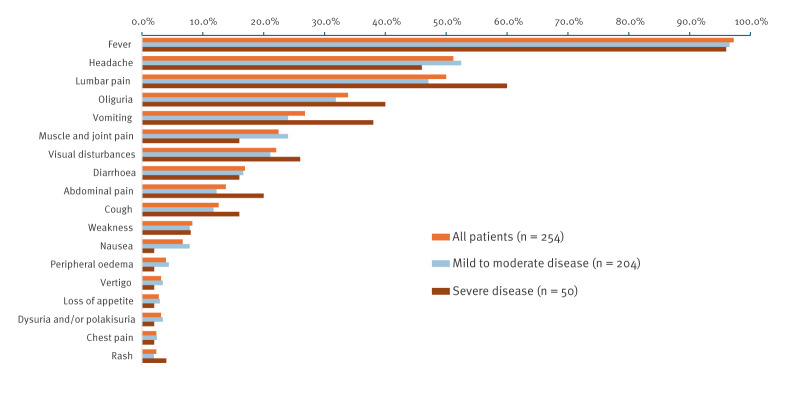
Symptoms of patients treated for hantavirus disease in the Clinical Hospital Center Rijeka, by disease severity, Croatia, 2021 (n = 254)

Of the 254 patients, 250 (98.4%) had symptoms or signs of an acute kidney disease on admission to the hospital (Figure 3). Acute kidney injury with an increase in serum creatinine ≥ 3 times the upper reference value (104 µmol/L in males, and 90 µmol/L in females, as defined for the Croatian population) [17] was found in 61 (24.0%) patients, 41 (23.2%) males and 20 (26.0%) females. Six of the patients needed haemodialysis. No bleeding disorders were noticed, besides microhaematuria, even in cases with the lowest thrombocyte counts (10 × 10^9^/L). We did not notice any new-onset arterial hypertension. Compared with mild to moderate disease, patients with severe HAVID had more often a systolic blood pressure < 100 mmHg on admission (OR = 3.10; 95% CI: 1.35–7.14; p = 0.004), abdominal symptoms, especially vomiting (OR = 2.02; 95% CI: 1.05–3.90; p = 0.018), leucocytosis (OR = 2.53; 95% CI: 1.34–4.78; p = 0.002), thrombocytopaenia (OR = 7.24; 95% CI: 0.96–54.69; p = 0.028), elevated urea levels (OR = 2.03; 95% CI: 1.04–3.95; p = 0.018) and CRP values ≥ 20 times above reference values (n = 23).

The re-evaluation of the chest radiographs of 155 (61.0%) patients confirmed pathological findings in 17: pleural effusion (n = 13), bronchopneumonia (n = 6; three with pleural effusions) and pulmonary oedema (n = 3; two with pleural effusions). During the first 2 months after HAVID onset, four patients experienced severe thrombotic or thromboembolic complications, including stroke, myocardial infarction, common femoral artery occlusion and pulmonary thromboembolism. One patient had a coinfection with Puumala virus and SARS-CoV-2 on admission, and four HAVID patients acquired COVID-19 during the hospital stay. These five patients had a severe form of HAVID. One HAVID patient developed respiratory failure and needed supplemental oxygen therapy after discharge. To our knowledge, the other patients recovered.

The challenging circumstances caused by the COVID-19 pandemic wave, due to reduced hospital capacities and the need of hospital admissions and isolation of COVID-19 patients, demanded the interdisciplinary management of most of the HAVID patients on an outpatient basis (n = 172; 67.7%), including day clinics. Thirty-three of the 50 cases with severe HAVID were treated as inpatients, and 49 of 204 of those with mild to moderate HAVID. Hospital admission rates were similar between male and female patients, and the median duration of in-patient treatment was 7 days (range: 3–42 days).

## Outbreak response measures

After the notification of HAVID cases to the IPH in March 2021, the epidemiologists of the regional IPHs alerted the GP practices of the endemic areas for HAVID via email on week 12. The public was informed about the increase in HAVID cases and the need to apply preventive and hygiene measures, with detailed instructions, by representatives of different specialties and sectors (e.g. infectious disease specialists, microbiologists, epidemiologists, forest companies, national parks) (Figure 2). The public was also informed about the possible overlap of symptoms and the difficulties of distinguishing HAVID from COVID-19. During April 2021, rodent extermination measures were intensified on the request of the residents and partly supported by the municipal authorities, for example in yards, barns, woodsheds and public surfaces (e.g. parks) in urban areas. Extensive measures were not possible because the Primorje-Gorski Kotar County and the Lika-Senj County have forests, nature and national parks where such interventions are not allowed. People living in the coastal areas of the Primorje-Gorski Kotar County, where no cases of hantavirus infection had been previously detected and notified, noticed a hitherto unprecedented infestation of rodents in their surroundings and contributed to raising awareness about the need of preventive measures by appealing via the media and competent communal services.

## Discussion

In 2021, the largest documented HAVID outbreak so far, caused by Puumala virus, occurred in the endemic regions of the northwestern inland of Croatia, in the Primorje-Gorski Kotar County and the Lika-Senj County [4]. During the previous decade, an average of 10 annual cases had been notified in the Primorje-Gorski Kotar County (population around 265,000 in 2021). In Europe, the highest incidence rates have been reported from Finland, for instance in 2016, 30.3 hantavirus infections per 100,000 population were notified [1]. In the outbreak we describe, the incidence rates in the affected regions were 72.3 per 100,000 population in the Primorje-Gorski Kotar County, and 138.0 per 100,000 population in the Lika-Senj County, while the incidence for the whole country was 8.6 cases per 100,000 inhabitants [4]. Of the 334 HAVID cases notified in Croatia, 275 (81.7%) were confirmed. Most cases were residing in the Primorje-Gorski Kotar County (n = 192; 57.5%) and in the Lika-Senj County (n = 59; 17.7%), primarily in the rural areas. However, we suspect an underdiagnosis and underreporting of suspected HAVID cases, as a substantial proportion of HAVID cases can remain undiagnosed because of oligo- or asymptomatic presentations [18]. Additionally, due to the COVID-19 pandemic, many patients unlikely contacted healthcare, and there was no outbreak investigation.

A cyclicity in the incidence of hantavirus infections has been observed in Croatia, usually related to increases in the rodent populations. The largest hantavirus outbreak was identified in 2017, with 389 cases (9.4/100,000 population), while 209 and 191 HAVID cases were notified in 2014 and 2019, respectively [1,4]. Simultaneously, outbreaks were reported in the neighbouring country of Slovenia, which is bordering Croatia along a large area with forests, and in Germany, Austria and France [1]. In the Balkan countries, most HAVID cases are seen in spring and summer, like in this outbreak, when the peak was in June. In the northern European countries, peaks in case numbers often coincide with vole peaks in late autumn and winter and exposure usually occurs via direct or indirect contact with bank vole excreta outdoors, in forestry cottages, sheds and farmhouses [1,8].

In this outbreak, for the first time, cases were seen among residents of the coastal region of the Primorje-Gorski Kotar County, where the Rijeka hospital is situated, the reference hospital for HAVID patients from the endemic areas of the two counties. Thirty patients from the coastal region had no history of visits or other possible exposure in known endemic areas for HAVID during the incubation period. Many patients noticed an increase in the number of rodents in the forests, as was described in earlier outbreaks in Croatia [5,6,11]. The increased notification rates subsided by the end of July, probably due to decreases in the rodent populations and thus risks of exposure [19]. In 2022, only six HAVID cases were notified in Croatia [4]. However, the virus persists in rodent populations, therefore, preventive measures are essential to minimise the risk of infection and future outbreaks [20].

Males dominated among the patients in this outbreak (177; 69.7% of the admitted patients), especially those aged < 45 years. According to ECDC data for 2020, the European notification rate was highest among those aged 45–64 years and hantavirus infection was more common in males, with an overall crude male-to-female ratio of 1.5:1 [1]. The male patients more often reported occupational exposure, while females predominantly were residents of endemic areas. In previous studies, close exposure to rodent habitats has been associated with hantavirus infections, such as occupation (agricultural or forestry workers, military personnel and zoologists) and other activities in endemic areas (cleaning of cottages, lying or sitting on the ground in forests, drinking water from forest springs, eating unwashed raw fruits harvested in the forest or hunting) [6,11,21-23].

Antibodies against Puumala virus were detected in 246 (96.9%) patients, and in the six cases of co-positivity (IgM Abs) against Puumala and Dobrava viruses, the antibody titre against Puumala virus was higher. This observation indicates cross-reactivity against these viruses that co-circulate in Croatia [11,12]. The simultaneous COVID-19 pandemic wave posed an additional challenge, due to the overlapping and often non-specific symptomatology of HAVID, the strain on healthcare system and the need to temporarily isolate all hospitalised patients until we received the SARS-CoV-2 PCR test results [24]. Difficulties in differentiating the clinical presentation of HAVID from COVID-19 have been reported [25,26].

Puumala virus infection is often a mild disease with a mortality rate of < 1% [1,13,27]. In our dataset, 50 (19.5%) patients had a severe form of HAVID, including one patient with HAVID-COVID-19 coinfection on admission and four HAVID patients who acquired COVID-19 during the hospital stay. We did not find any significant difference in disease severity based on exposure. Only three teenagers aged < 18 years were diagnosed with HAVID and no cases in younger minors. This is in line with the low hantavirus infection rates in children in the ECDC data, e.g. in 2020, < 0.04 cases per 100,000 of the total population [1]. Our patients had no history of a prior hantavirus infection, and we did not find any reports in the literature of reinfection with this virus [28]. Immunity after HAVID usually lasts for many years [3,9,18,29], which could explain the lower proportion of male patients with higher age.

None of the patients in our study had petechiae or bleeding episodes, contrary to the earlier observations in HAVID caused by Puumala virus [22,30,31], and among the patients with a severe course, only two had high haemoglobin concentrations. Elevated haemoglobin/haematocrit concentrations were identified as hallmarks of the more severe form of hantavirus disease HCPS [32]. However, we noticed thrombotic or thromboembolic complications in four patients, as has been described in the literature [33,34]. Of the 50 patients with severe HAVID, 23 had elevated CRP values ≥ 20 times above the reference values, indicating a strong inflammatory response and often resulting in the introduction of empirical antimicrobial treatment. In some other studies, higher CRP values have not been associated with a more severe HAVID [35].

Severe acute kidney injury, with an increase in serum creatinine ≥ 3 times the upper reference value, developed in 61 (24.0%) patients, with a similar proportion in males and females, and six of them needed haemodialysis treatment. Immune-mediated effects, as well as viral infection of kidney cells, contribute to kidney damage, as reported in the literature [36]. During the first presentation, a higher proportion of patients with severe HAVID had a systolic blood pressure < 100 mmHg than those with mild to moderate disease (OR = 3.10; 95% CI: 1.35–7.14; p = 0.004), which probably was related to a more severe inflammatory response and vascular leakage, as the infection primarily targets the endothelial cells [27,37]. We did not notice any new-onset arterial hypertension, although this has been seen in other reports [11,27]. Abdominal symptoms, especially vomiting, were more frequent in patients with severe compared with mild to moderate HAVID (OR = 2.02; 95% CI: 1.05–3.90; p = 0.018), as is seen in other haemorrhagic fevers such as dengue [38]. Hantavirus disease may be associated with respiratory symptoms, interstitial pulmonary infiltrations and pleural effusions [11]. Chest radiographs were performed in 156 (60.9%) of our patients, and we noticed HAVID-associated changes in 17 (6.6%) of them, as pleural effusion, bronchopneumonia or pulmonary oedema, but no case with interstitial pneumonia. All patients had recovered when discharged from the hospital or by the end of the treatment in the day clinic, except for one patient needing oxygen treatment.

Main limitations of our study are the lack of a robust outbreak investigation and collection of more comprehensive epidemiological data and that no analytical epidemiological methods were used. As the study was based on patients treated in the hospital, as inpatients or in the day clinic, we do not know the total number of cases.

To prevent infections, joint action is necessary to ensure efficient measures [1,3], such as (i) control and prevention of the expansion of rodent populations by regular rodent control measures in urban areas according to the regulations; (ii) adherence to general hygiene measures, wearing masks and gloves during activities with the risk of exposure, such as cleaning and working in barns, woodsheds or basements, gathering firewood, farming and agricultural activities; (iii) raising awareness among the healthcare personnel and public of this viral disease, primarily in persons living in, working in or travelling to hantavirus endemic areas; (iv) testing suspected cases and notifying every case to the public health authorities to enable early recognition of outbreaks and the implementation of efficient prevention.

## Conclusion

Early clinical suspicion of HAVID, timely diagnosis with the support of infectious disease specialists and nephrologists, the rapid implementation of advanced supportive treatment, interdisciplinary collaboration and patient follow-up are crucial for improving outcomes. The outbreak emphasised the importance of rising awareness, notification of cases and implementing effective measures, also in areas with no previous cases.
